# A new T classification based on masticator space involvement in nasopharyngeal carcinoma: a study of 742 cases with magnetic resonance imaging

**DOI:** 10.1186/1471-2407-14-653

**Published:** 2014-09-04

**Authors:** Dong-Hua Luo, Jing Yang, Hui-Zhi Qiu, Ting Shen, Qiu-Yan Chen, Pei-Yu Huang, Rui Sun, Chao-Nan Qian, Hai-Qiang Mai, Xiang Guo, Hao-Yuan Mo

**Affiliations:** State Key Laboratory of Oncology in South China; Collaborative Innovation Center for Cancer Medicine, Sun Yat-sen University Cancer Center, Guangzhou, PR China; Department of Nasopharyngeal Carcinoma, Sun Yat-sen University Cancer Center, 651 Dongfeng Road East, Guangzhou, Guangdong 510060 PR China; Department of Radiotherapy, Affiliated Cancer Center, Guangzhou Medical College, Guangzhou, Guangdong 510095 PR China

**Keywords:** Nasopharyngeal carcinoma, Masticator space involvement, Magnetic resonance imaging, Prognosis

## Abstract

**Background:**

The aim of this study was to investigate the prognostic significance and various classifications for anatomic masticator space involvement (MSI) in patients with nasopharyngeal carcinoma (NPC).

**Methods:**

This study retrospectively analyzed 742 patients with untreated nondisseminated NPC who underwent magnetic resonance imaging (MRI) scan of the nasopharynx and neck. The MSI was graded according to different anatomic features. The overall survival (OS), local relapse-free survival (LRFS), distant metastasis-free survival (DMFS), and disease-free survival (DFS) of the patients with different MSI grades were analyzed using the Kaplan-Meier method and log-rank tests.

**Results:**

The frequency of MSI was 24.1% (179/742). The 5-year OS, LRFS, DMFS, DFS for NPC patients with versus without MSI were 70.9% versus 82.5% (*P =* 0.001), 94.1% versus 91.4% (*P* = 0.511), 81.4% versus 88.7% (*P* = 0.021), and 78.0% versus 83.5% (*P =* 0.215), respectively. Significant differences in OS were also found among different MSI groups. In the patients with MSI, the OS of the group with medial and/or lateral pterygoid involvement (MLPI) NPC was 73.9% compared to 51.3% (*P <* 0.0001) in the patients with infratemporal fossa involvement (IFI).

**Conclusions:**

MSI was an independent prognostic factor for OS and DMFS. NPCs invading the masticator space should be separately categorized into MLPI and IFI prognostic groups. We suggest that MLPI should be staged as T3 while IFI is staged as T4 disease in future TNM staging revision.

## Background

Nasopharyngeal carcinoma (NPC) is one of the most common malignant tumors in southern China and Southeast Asia with incidences reported as 15-50 per 100,000 in high-incidence areas [1–7].

**Figure 1:**
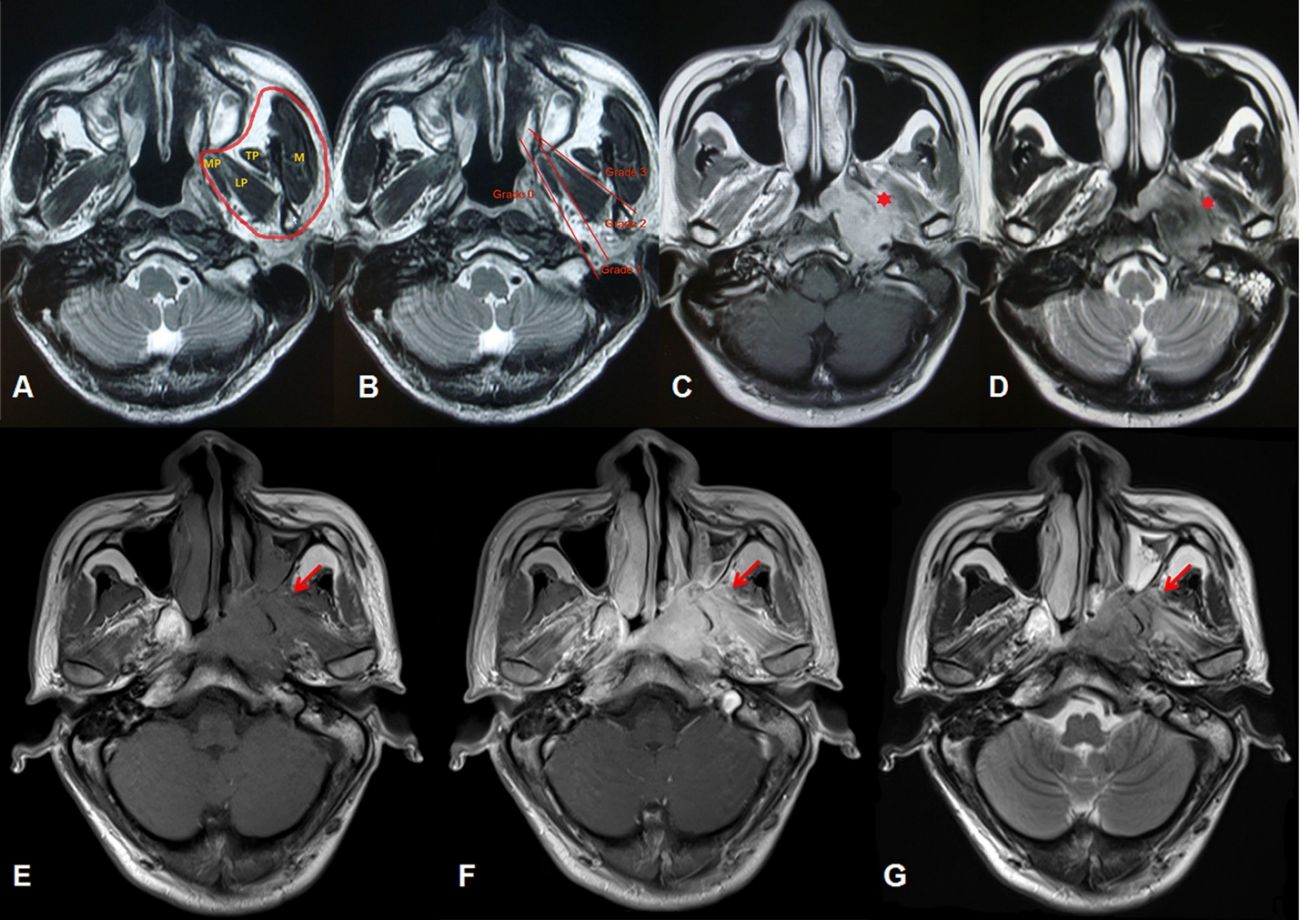
**Normal masticator space and different grades of masticator space involvement in magnetic resonance images. (A)** Axial T2-weighted magnetic resonance (MR) image (1800 ~ 3000 ms/90 ~ 150 ms, TR/TE) at the level of the nasopharynx shows the anatomic masticator space (circled in red). LP = lateral pterygoid muscle, M = masseter muscle, MP = medial pterygoid muscle, TP = temporalis muscle. **(B)** Grades of masticator space involvement. Grade 0: without MSI; Grade 1: with medial pterygoid muscle involvement but without lateral pterygoid muscle involvement or infratemporal fossa involvement; Grade 2: with medial and /or lateral pterygoid muscle but without infratemporal fossa involvement; Grade 3: with infratemporal fossa involvement. **(C)** T1-weighted axial contrast medium–enhanced MR image and **(D)** T2-weighted MR image show extensive tumor infiltration in the left masticator space. Medial and lateral pterygoid muscle involvement is marked with a red asterisk in each image. **(E)** T1-weighted axial MR image, **(F)** T1-weighted axial contrast medium–enhanced MR image, and **(G)** T2-weighted MR image show left infratemporal fossa involvement (red arrow).

NPC is an aggressive disease and tends to involve surrounding tissues and organs. The masticator space is one of the most vulnerable structures. Anatomically, the masticator space is defined as a deep facial space enclosed by the superficial layer of deep cervical fasciae, which is located in the anterior-lateral side of the parapharyngeal space. It contains four muscles of mastication: the medial and lateral pterygoid muscles, the masseter muscle and the temporalis muscle. The content of the masticator space also includes the additional structures encompassed within these fascial boundaries. These structures include the ramus of the mandible and the third division of the fifth cranial nerve (CN V) as it passes through the foramen ovale into the suprahyoid neck [8–10] (Figure 1A). The inferior limit of the anatomic masticator space is the attachment of the medial pterygoid muscle to the mandible, whereas the superior limit is the base of the skull [8, 9]. The entire masticator space can be divided into the supratemporal fossa and intratemporal fossa by the zygomatic arch, the latter of which is known as an inherent part of the masticator space. The masticator space plays an important role in the tumor staging system of NPC. Radiology textbooks often use the same definition of “masticator space” with inclusion of the medial and lateral pterygoid muscles [11].

Currently, the Chinese 2008 staging system [12] and the seventh edition American Joint Committee on Cancer staging system (AJCC 7th, 2009) [13] are commonly used in China and abroad. These two new staging systems possess certain similarities and differences [14]. One of the major differences is varying T stage for masticator space involvement. The most ambiguous term among the defining criteria is “masticatory space”. This was introduced in the 6th edition as a synonym of infratemporal fossa, defined as extension beyond the anterior surface of the lateral pterygoid muscle or beyond the posterolateral wall of the maxillary antrum and/or the pterygo-maxillary fissure [15]. Unfortunately, this differs from the definition used in classical radiological textbooks as “primarily the muscles of mastication (the medial and lateral pterygoid, masseter and temporalis) enclosed by the superficial layer of the deep cervical fascia”, and this description was adopted in the 7th edition [13, 16]. The study by Tang et al. [10] supported this definition for T4 classification in AJCC 7th edition due to its significant impact on the overall survival and local relapse-free survival of patients with NPC. As a result, the authors recommended that anatomic masticator space involvement including the medial and lateral pterygoid muscles be classified as stage T4 disease. According to their results, tumors with extension limited to adjacent pterygoid muscles could be over-staged and classified as T4. However, these tumors generally have a much better prognosis, and incorrect staging may lead to potentially unnecessary treatment. In the Chinese 2008 system, medial and lateral pterygoid muscles were included in the definition of masticator space, and masticator space involvement excluding medial pterygoid muscles was classified as T4, while medial pterygoid involvement was classified as T3 [12].

This study retrospectively analyzed 742 patients with untreated nondisseminated NPC who underwent MRI scan of the nasopharynx and neck. The MSI was graded according to different anatomic features. By comparing our data with established staging systems, we aimed to establish an optimal grading method for masticator space involvement and determine the prognostic value to facilitate treatment strategies in patients with NPC. The medial and/or lateral pterygoid involvement is abbrievated as MLPI and infratemporal fossa (as definition in the 6th edition) involvement is abbrievated as IFI.

## Methods

### Patients

We reviewed the records of consecutive NPC patients referred to Sun Yat-sen University Cancer Center between January 1, 2005 and December 31, 2005 with histologically proven NPC without distant metastasis. The cohort consisted of 575 male and 167 female patients, giving a male: female ratio of 3.44:1. The median patient age was 46 y (range 16–78 y). Histologically, 717 (96.63%) patients had World Health Organization (WHO) Type III disease, and 25 (3.37%) had WHO Type II disease. Table 1 shows the characteristics of all patients.

**Table 1:** **Patient characteristics categorized by MSI (N = 742)**

	N = 742	Without MSI	With MSI n =180			***P***Value
		0(n = 562)	1(n = 119)	2(n = 38)	3(n = 23)	
**Age(Y)**						0.397
**<46**	367	279	61	19	8	
	(49.46%)	(49.64%)	(51.26%)	(50.00%)	(34.78%)	
**> = 46**	375	283	58	19	15	
	(50.54%)	(50.36%)	(48.74%)	(50.00%)	(65.22%)	
**UICC 7th T**						*<*0.001
**T1, T2**	308	292	15	0	0	
	(41.51%)	(51.96%)	(12.61%)	(0)	(0)	
**T3, T4**	434	270	104	38	23	
	(58.49%)	(48.04%)	(87.39%)	(100%)	(100%)	
**UICC 7th N**						0.047
**N0, N1**	554	413	89	32	20	
	(74.66%)	(73.49%)	(74.79%)	(84.21%)	(86.96%)	
**N2, N3**	188	149	30	6	3	
	(25.34%)	(26.51%)	(25.21%)	(15.79%)	(13.04%)	
**Chemotherapy**						<0.001
**No**	200	184	13	1	2	
	(26.95%)	(32.74%)	(10.92%)	(2.63%)	(8.70%)	
**Induction**	217	143	42	24	8	
	(29.25%)	(25.44%)	(35.29%)	(63.16%)	(34.78%)	
**Induction + CCRT**	112	69	27	8	8	
	(15.09%)	(12.28%)	(22.69%)	(21.05%)	(34.78%)	
**Induction + Adjuvant**	9	8	1	0	0	
	(1.21%)	(1.42%)	(0.84%)	(0)	(0)	
**CCRT**	171	132	32	4	3	
	(23.05%)	(23.49%)	(26.89%)	(10.53%)	(13.04%)	
**CCRT + Adjuvant**	16	13	2	0	1	
	(2.16%)	(2.31%)	(1.68%)	(0)	(4.35%)	
**Induction + CCRT + Adjuvant**	16	12	2	1	1	
	(2.16%)	(2.14%)	(1.68%)	(2.63%)	(4.35%)	
**Adjuvant**	1	1	0	0	0	
	(0.13%)	(0.18%)	(0)	(0)	(0)	

This retrospective study was approved by the Clinical Research Ethics Committee of the Sun Yat-sen University Cancer Centre, and all the participants provided written informed consent before treatment.

### Pretreatment evaluation

The pretreatment patient evaluation included a complete medical history, physical and neurologic examinations, hematological studies, and biochemical profiles. All patients underwent fiberoptic endoscopy of the nasopharynx, oropharynx and larynx and were examined with magnetic resonance imaging (MRI) of the nasopharynx and the neck. Biopsies of all primary tumors for histologic diagnosis were performed for all patients before treatment. The metastatic workup included chest radiographs, abdominal sonography, and a whole body bone scan using single photon emission computed tomography (SPECT) or positron emission tomography-computed tomography (PET/CT). All patients’ clinical stages were reclassified according to the AJCC 7th edition staging system.

### MR imaging protocol

All patients underwent MRI with a 1.5 T system (Singa Excite/or HDX 1.5 T, American GE Company). The MRI was performed on spiral echo (SE) sequence, with scanning directions of cross section, sagittal plane, and coronal plane. The area from the suprasellar cistern to the inferior margin of the sterna end of the clavicle was examined with a head-and-neck combined coil in a slice thickness of 5 mm with 0.5 mm interslice gap. The following MRI sequences were applied: T1-weighted spin echo images (400–600 ms/15 ~ 25 ms TR/TE), T2-weighted fast spin echo images (1800 ~ 3000 ms/90 ~ 150 ms, TR/TE), and enhanced T1-weighted spin echo images with gadolinium-DTPA (Gd-DTPA) injection at a dose of 0.1 mmol/kg body weight.

### Image assessment and grades of MSI

All MRI images were reviewed to minimize heterogeneity in restaging. Two radiologists specialized in head and neck cancers independently evaluated all scans. Any disagreements were resolved by consensus.

The presence of MSI was defined based on MRI findings and by the presence of low-density signal on T1-weighted images, high signal changes on T2-weighted images, and enhancement by Gd-DTPA in the masticator space complex. As described above, the masticator space complex includes the medial and lateral pterygoid muscles, the masseter muscle, the temporalis muscle, and any spaces between them. A diagnosis of MSI is made if the muscle is indistinguishable from the tumor mass by signal intensity, if asymmetry in signal intensity exists, or if the integrity of the muscles of mastication has been disrupted by the tumor in two orthogonal views (Figure 1C, D).

Patients without MSI were recorded as grade 0. Patients with medial pterygoid muscle involvement but without lateral pterygoid muscle involvement or infratemporal fossa involvement recorded as grade 1. Patients with lateral pterygoid muscle involvement but without infratemporal fossa involvement recorded as grade 2, and any infratemporal fossa involvement (IFI) was recorded as grade 3 (Figure 1B).

### Patient treatment

All patients received radical radiotherapy. Two different techniques were applied for the patients in different TNM stages. In this study, 83.6% (620/742) of patients received two-dimensional conformal radiotherapy, and 16.4% (122/742) received three-dimensional conformal radiotherapy (3D-CRT) or intensity-modulated radiotherapy (IMRT). The patients at early stages (stages I and II) were treated using radiotherapy alone. The patients at advanced stages (stages III and IV) received radiotherapy and combined neoadjuvant chemotherapy and/or concurrent chemotherapy. The techniques of low melting-point lead block, multi-leaf collimator (MLC), thermoplastic mask and source axis distance (SAD) were applied to radiotherapy. Cobalt-60 (Co-60) gamma-rays or 6-8 MV supervoltage X rays generated by a linear accelerator were used for external irradiation. The fraction of two-dimensional conformal radiotherapy was conventional (2 Gy/F, 5 F/Week), and the dose for the primary lesion in the nasopharynx was 66 ~ 76 Gy/33 ~ 38 F. The dose for the cervical lymphatic drainage area was 50 ~ 66 Gy/25 ~ 33 F. Cobalt-60 (Co-60) gamma-rays or supervoltage X ray added beta-rays were used to compensate the dose in consideration of skin and subcutaneous tissues in the neck. The prescribed radiation doses of 3D-CRT were defined as follows [17]: GTVnx (nasopharynx gross tumor volume): 65-70 Gy; GTVnd (positive neck lymph nodes volume): 60-70 Gy; CTV60 (clinical target volume 60): 60 Gy; CTVnx50 (nasopharynx clinical target volume 50): 50 Gy;CTVnd50 (neck nodal clinical target volume): 50 Gy. The prescribed radiation dose of IMRT was defined as follows [18, 19]: a total dose of 68 Gy in 30 fractions at 2.27 Gy per fraction to the planning target volume (PTV) of the primary gross tumor volume (GTV-P), 60 to 64 Gy to the PTV of nodal gross tumor volume (GTV-N), 60 Gy to the PTV of CTV-1 (i.e., high-risk regions), and 54 Gy to the PTV of CTV-2 (i.e., low-risk regions) and CTV-N (i.e., neck nodal regions). The treatment was delivered by a dynamic, multileaf, intensitymodulating collimator (called MIMiC). For the lower neck, an anterior cervical field was used. All patients were treated with one fraction daily over 5 days per week. The patients with residual tumor confined in the nasopharyngeal cavity after external irradiation would receive after-loading irradiation of 2-3 F, 5 Gy/F. In this study, 73% (542/742) of patients received platinum-based neoadjuvant, concurrent, or adjuvant chemotherapy.

### Patient follow-up

The duration of patient follow-up was calculated from the first day of treatment to either the day of death or the day of the last examination. The patients were examined at least every 3 months during the first 2 years; thereafter, a follow-up examination (including nasopharyngoscopy, MRI of the head and neck, chest radiography, and abdominal sonography) were performed every 6 months for up to 5 years or until death.

All events were measured from the date of treatment commencement. The following end points (time to the first defining event) were assessed: overall survival (OS); local relapse-free survival (LRFS); distant metastasis-free survival (DMFS); and disease-free survival (DFS). The OS was defined as the duration from the date of each patient’s treatment commencement to the date of death from any cause or the censoring of the patient at the date of the last follow-up. The DFS was defined as the duration from the date of treatment commencement to the date of disease progression of the patient at the date of the last follow-up. The LRRFS and DMFS were also evaluated and calculated from the date of each patient’s treatment commencement until the day of the first locoregional or distant relapse or until the date of the last follow-up visit. Local recurrence was determined by endoscopy and biopsy or MRI. Distant metastases were diagnosed on the basis of clinical symptoms, physical examination, and imaging methods, including chest radiography, bone scans, MRI and abdominal sonography. As described in our previous paper [20].

### Statistical analysis

The Statistical Package for Social Sciences, version 16.0 (SPSS Inc., Chicago, IL) was used. Actuarial rates were calculated using the Kaplan–Meier method and differences were compared using the log-rank test. Multivariate analyses using the Cox proportional hazards model were used to calculate the hazard ratio (HR). The Cox proportional hazards model was also used to test hazard consistency and hazard discrimination. Host factors (age and sex) and T classification were included as covariates in all tests. A two-tailed *P* value less than 0.05 was considered statistically significant.

## Results

### Follow-up outcomes

The time of last follow-up was July 2010, and the median follow-up period was 63.6 months. Of all 742 patients, 57 developed locoregional relapse including 47 cases of nasopharyngeal relapse and 10 cases of cervical lymph node relapse. 92 patients developed distant metastasis including 21 cases of bone metastasies, 15 cases of liver metastasis, 13 cases of lung metastasis, 41 cases of multi-organ metastasis, 1 case of auxiliary lymph node metastasis and 1 case of vertebral canal metastasis. The 5-year survival rates were as follows: OS, 80.5%; DFS, 82.1%; LRFS, 92.3%; DMFS, 87.6%.

### Patient characteristics categorized by masticator space involvement

The incidence of MSI was 24.1% (179/742) and included 118 (15.9%) cases of grade 1, 38 (5.1%) cases of grade 2, and 23 (3.1%) cases of grade 3 disease. Table 1 shows a comparison of the patient characteristics, T and N classification, chemotherapy, and presence of MSI. A significantly higher proportion of patients with MSI had advanced T stage (*P <* 0.001). A correlation was also observed between MSI and N stage (*P =* 0.047). There was also correlation between chemotherapy strategy and MSI (*P <* 0.001).

### Masticator space involvement associated with more aggressive tumor extension

Univariate analysis showed masticator space involvement was associated with intracranial extension, tumor invasion of prevertebral space, base of skull bony structure, paranasal sinuses and parapharyngeal space (Table 2).

**Table 2:** **Association of primary tumor extension and MSI**

Primary tumor extension	MSI -		MSI +		
	No.	%	No.	%	***P***value
**Intracranial**	28	5.0	56	31.1	<0.001
**Prevertebral space**	213	38.0	156	86.7	<0.001
**Base of skull bony structure**	260	46.3	159	88.3	0.002
**Paranasal sinuses**	37	6.5	52	28.9	0.020
**Parapharyngeal space**	334	59.4	158	87.8	0.045

### Masticator space involvement in general is an independent prognostic factor for OS and DMFS

**Figure 2:**
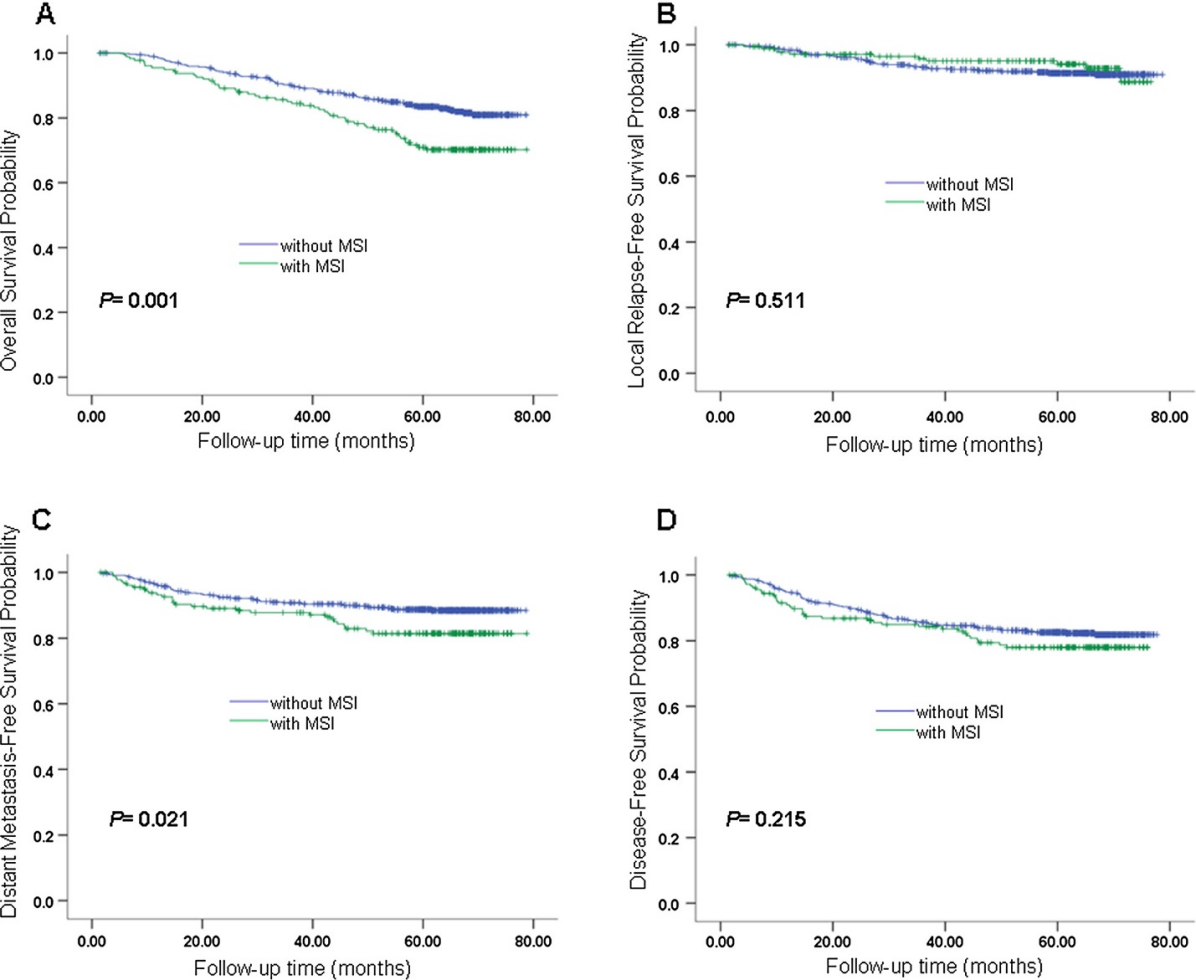
**Survival analyses for MSI. (A)** Overall survival, **(B)** local relapse-free survival, **(C)** metastasis-free survival and **(D)** disease-free survival for patients with MSI and patients without MSI. MSI, masticator space involvement.

The 5-year OS for NPC patients with and without MSI was 70.9% and 82.5%, respectively (P = 0.001; Figure 2A). The 5-year LRFS for patients with and without MSI was 94.1% and 91.4%, respectively (P = 0.511; Figure 2B). The 5-year DMFS was 81.4% and 88.7% for patients with and without MSI, respectively (P = 0.021; Figure 2C). The 5-year DFS was 78.0% and 83.5% for patients with and without MSI, respectively (P = 0.215; Figure 2D).

To verified the effect of MSI in patients with 3D-CRT/IMRT. Patients treated with 3D-CRT/IMRT and patients treated with conventional radiotherapy had been analyzed separately as following: (1) There were 122 cases totally in 3D-CRT/IMRT population, 26 with MSI and 96 without MSI, giving an approximate ratio of 1:3. OS, LRFS, DMFS and DFS of MSI group and non-MSI group were estimated by Kaplan-Meier analysis and the differences of the survival probabilities were compared by Log-rank test. The results showed statistically significant differences in OS (P < 0.001), DMFS (P = 0.005) and DFS (P = 0.017), while not in LRFS (P = 0.282). (2) There were 620 cases totally in conventional radiotherapy population, 154 with MSI and 466 without MSI, also giving an approximate ratio of 1:3, just similar with 3D-CRT/IMRT population. The same analyses were applied, results showed statistically significant differences in OS (P < 0.001), DMFS (P = 0.005) and DFS (P = 0.008), while not in LRFS (P = 0.639). Overall, compared with patients without MSI, patients with MSI had a worse prognosis for OS and DMFS by univariate analysis.

Several parameters were included in the Cox proportional hazards model, and multivariate analysis was performed to adjust for various prognostic factors. The included parameters were the following: age (≤46 y vs. >46 y), gender (female vs. male), WHO histological grade (Type II vs. III), T stages (T1-2 vs. T3-4), N stages (N0-1 vs. N2-3), chemotherapy (with vs. without), radiation therapy technique (two-dimensional conventional radiation therapy vs. three-dimensional conformal radiation therapy vs. intensity-modulated radiation therapy), and MSI (with vs. without). The OS, LRFS, DMFS and DFS were evaluated as endpoints. T stage has a positive correlation with MSI (R = 0.379, *P <* 0.001), and the correlation is significant at the 0.01 level (2-tailed). T stages will cover MSI when they are both included in multivariate analysis. Therefore, we analyzed these parameters separately. When T stage was included with MSI the independent prognostic factors for OS were age, T stage and N stage. The independent prognostic factor for LRFS was T stage. The T stage and N stage were also independent prognostic factors for both DMFS and DFS. When MSI was included without T stages, the independent prognostic factors for OS were gender, age, N stage and MSI. Both MSI and N stage were independent prognostic factors for DMFS and N stage was an independent prognostic factor for DFS (Table 3). Thus, MSI was an independent prognostic factor for OS and DMFS.

**Table 3:** **Multivariate analysis of prognostic factors for NPC patients**

Endpoint and Variable	Factors	***P***value	Odds ratio*
**Death**	Gender (female VS. male)	0.042	0.629(0.402,0.984)
	Age (≤46 y VS >46 y)	<0.0001	1.837(1.313,2.570)
	MSI (with VS. without)	<0.002	1.770(1.234,2.539)
	N classification (N0-1 VS N2-3)	<0.0001	2.035(1.428,2.901)
**Local regional failure**	Gender (female VS. male)	0.052	0.455(0.206,1.006)
**Distant failure**	MSI (with VS. without)	0.027	1.658(1.058,2.596)
	N classification (N0-1 VS N2-3)	<0.0001	2.828(1.849,4.326)
**Disease failure**	N classification (N0-1 VS N2-3)	<0.001	2.094(1.460,3.002)

*Data in parentheses are 95% confidence intervals.

### Involvement of infratemporal fossa was an unfavorable independent prognostic factor

Whether different MSI involvement grade has prognostic value is still not clear. We further investigated if there is a more appropriate classification for MSI in terms of its prognostic value. According to the different grades of MSI, three categories were listed by permutation and combination:

**Classification pattern A:** grade 0 converted to PA0, grade 1 converted to PA1, grade 2 converted to PA2, grade 3 converted to PA3.

**Classification pattern B:** grade 0, grade 1 and grade 2 converted to PB0, grade 3 converted to PB1.

**Classification pattern C:** grade 0 converted to PC0, grade 1 and grade 2 converted to PC1, grade 3 converted to PC2.

**Figure 3:**
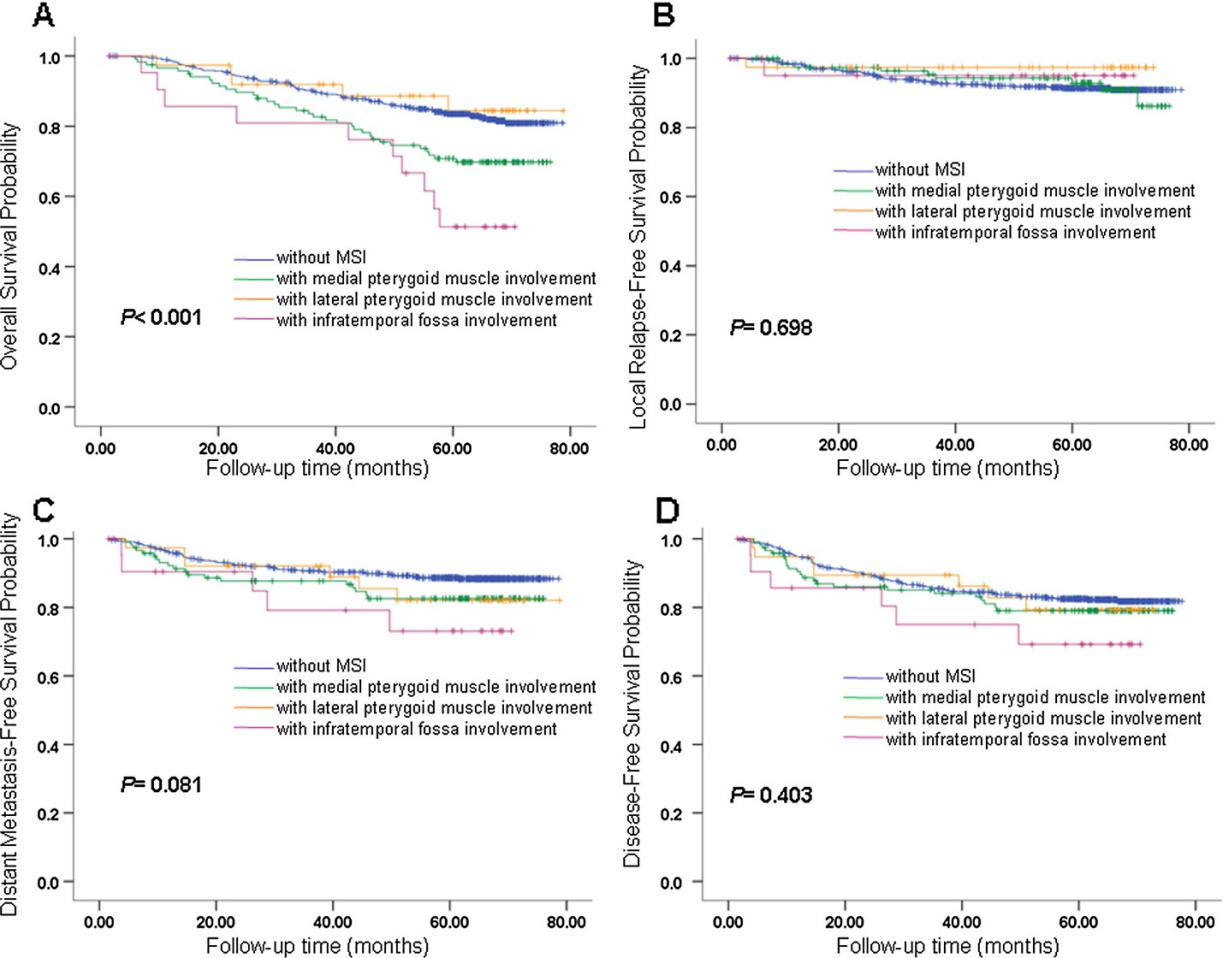
**Survival analyses for MSI Pattern A. (A)** Overall survival, **(B)** local relapse-free survival, **(C)** metastasis-free survival and **(D)** disease-free survival for different groups of MSI in pattern A. MSI, masticator space involvement. Classification pattern A: grade 0 converted to PA0, grade 1 converted to PA1, grade 2 converted to PA2, grade 3 converted to PA3. Grades of masticator space involvement. Grade 0: without MSI; Grade 1: with medial pterygoid muscle involvement but without lateral pterygoid muscle involvement or infratemporal fossa involvement; Grade 2: with medial and/or lateral pterygoid muscle but without infratemporal fossa involvement; Grade 3: with infratemporal fossa involvement.

**Figure 4:**
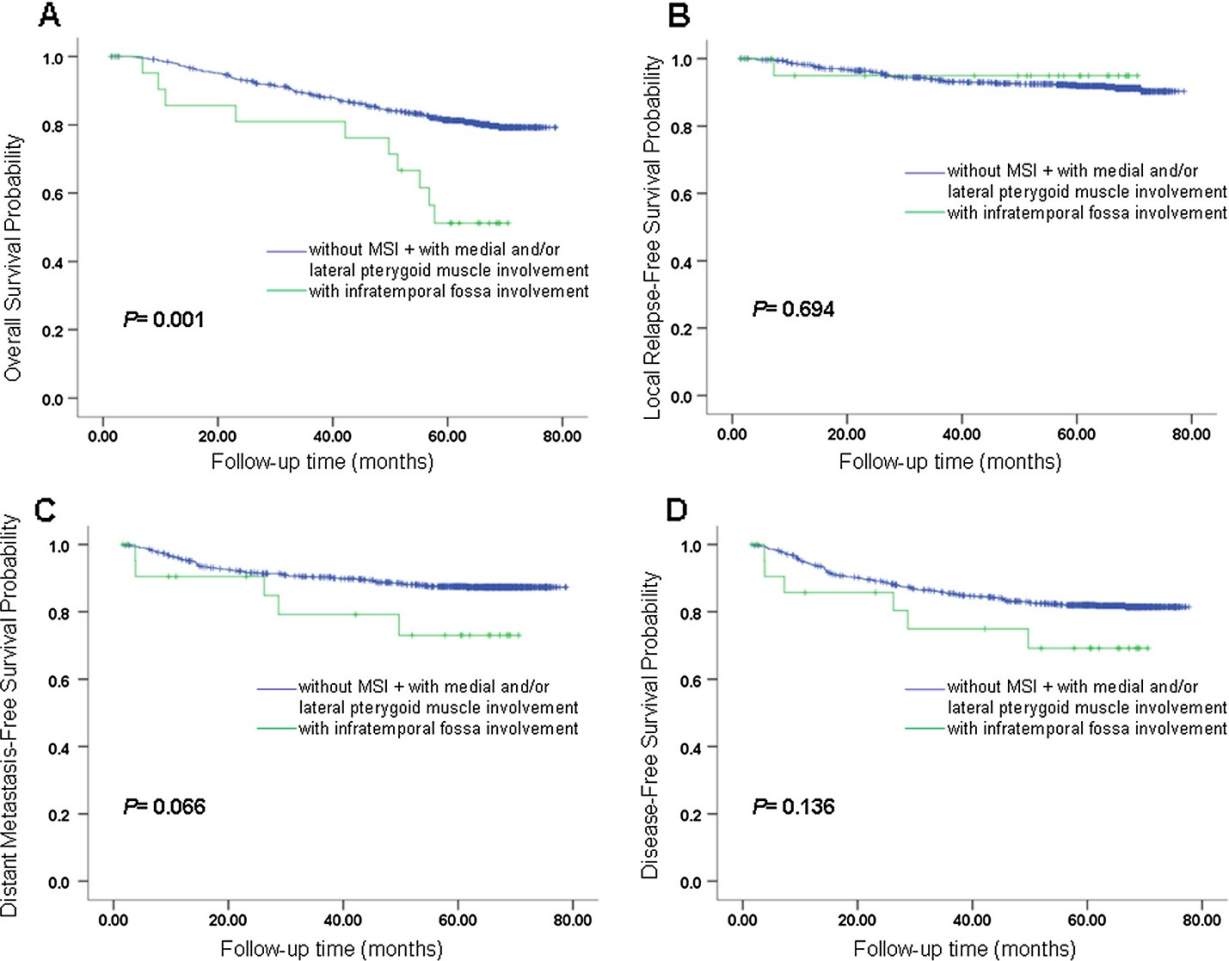
**Survival analyses for MSI Pattern B. (A)** Overall survival, **(B)** local relapse-free survival, **(C)** metastasis-free survival and **(D)** disease-free survival for different groups of MSI in pattern B. MSI, masticator space involvement. Classification pattern B: grade 0, grade 1 and grade 2 converted to PB0, grade 3 converted to PB1. Grades of masticator space involvement. Grade 0: without MSI; Grade 1: with medial pterygoid muscle involvement but without lateral pterygoid muscle involvement or infratemporal fossa involvement; Grade 2: with medial and /or lateral pterygoid muscle but without infratemporal fossa involvement; Grade 3: with infratemporal fossa involvement.

**Figure 5:**
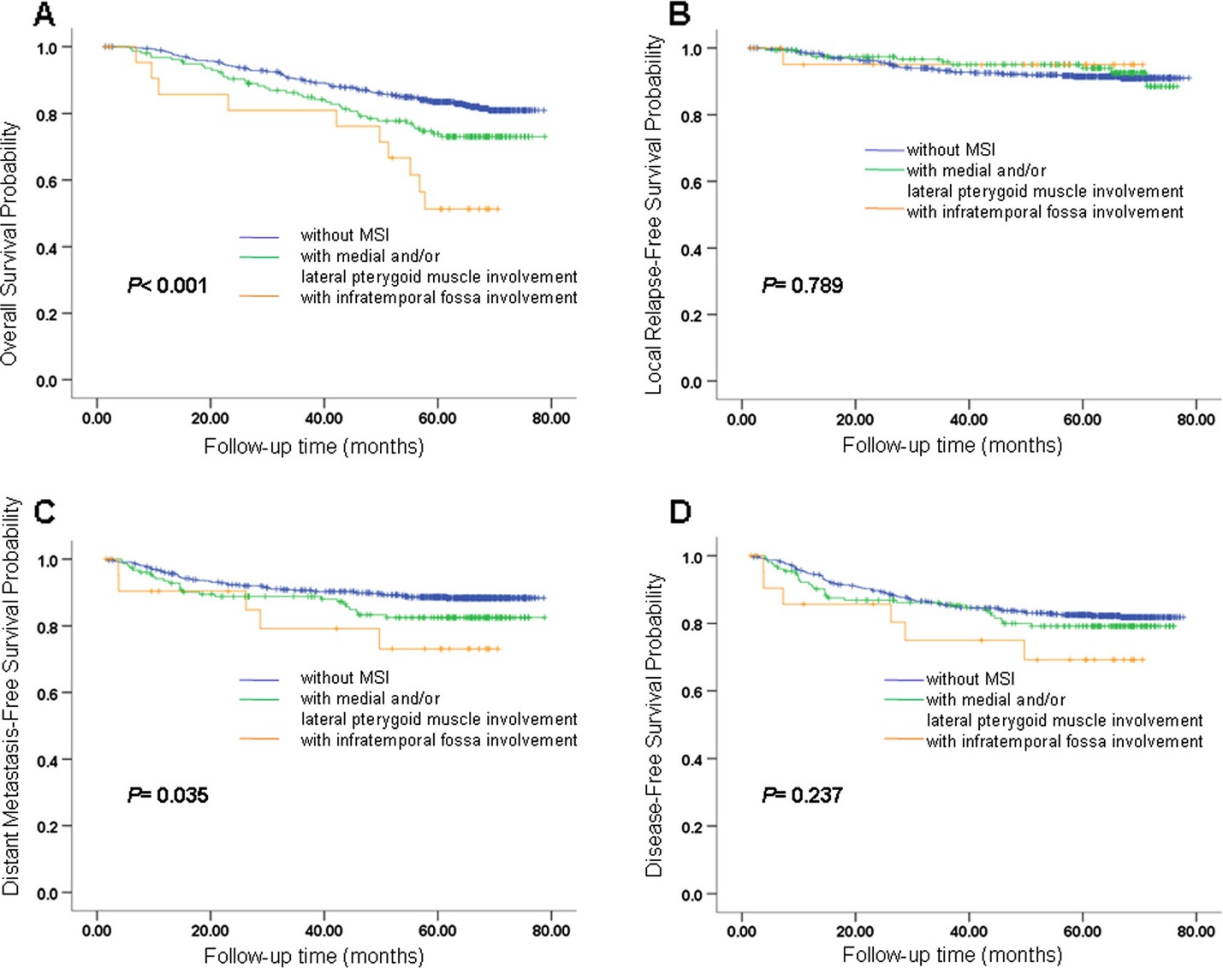
**Survival analyses for MSI pattern C. (A)** Overall survival, **(B)** local relapse-free survival, **(C)** metastasis-free survival and **(D)** disease-free survival for different groups of MSI in pattern C. MSI, masticator space involvement. Classification pattern C: grade 0 converted to PC0, grade 1 and grade 2 converted to PC1, grade 3 converted to PC2. Grades of masticator space involvement. Grade 0: without MSI; Grade 1: with medial pterygoid muscle involvement but without lateral pterygoid muscle involvement or infratemporal fossa involvement; Grade 2: with medial and /or lateral pterygoid muscle but without infratemporal fossa involvement; Grade 3: with infratemporal fossa involvement.

Multivariate analysis was applied using a Cox regression model in each MSI classification pattern to identify prognostic factors for OS, LRFS, DMFS and DFS. The analyses revealed that classification patterns A, B and C showed MSI grade was an independent prognostic factor for OS (*P <* 0.041 in pattern A, *P =* 0.018 in pattern B, *P =* 0.007 in pattern C), but not for LRFS, DMFS and DFS. The other independent prognostic factors were not changed and these factors are presented in Table 4. The OS, LRFS, DMFS, and DFS curves for different groups of MSI in patterns A, B and C are shown in Figures 3, 4 and 5. Based on the Cox regression model in pattern A, it was suggested that each grade of MSI might influence OS of the patients. Both classification patterns B and C indicate that involvement of infratemporal fossa (IFI) had a worse OS than involvement of medial pterygoid and/or lateral pterygoid (MLPI). In pattern C, we also found that IFI has a worse DMFS than MLPI (*P =* 0.035). In patterns A and B, we also found a similar trend (*P =* 0.081 in pattern A, *P =* 0.066 in pattern B).

**Table 4:** **Multivariate analysis of prognostic factors for NPC patients in pattern A, B, C**

	OS		LRFS		DMFS		DFS	
Patterns/Variables	***P***value	Hazard ratio (95% CI)	***P***value	Hazard ratio (95% CI)	***P***value	Hazard ratio (95% CI)	***P***value	Hazard ratio (95% CI)
**Pattern A**								
**Age**	<0.001	-			<0.001	-	<0.001	-
**T stage**	<0.001	-	0.004	-	<0.001	-	<0.001	-
**N stage**	<0.001	-						
**MSI grade**	0.041*	(1.162,4.402)	0.258		0.394		0.638	
**Pattern B**								
**Age**	<0.001	-			<0.001	-	<0.001	-
**T stage**	<0.001	-	<0.001	-	<0.001	-	<0.001	-
**N stage**	<0.001	-						
**MSI grade**	0.018*	(1.148,4.226)	0.504		0.112		0.273	
**Pattern C**								
**Age**	<0.001	-			<0.001	-	<0.001	-
**T stage**	<0.001	-	0.004	-	<0.001	-	<0.001	-
**N stage**	<0.001	-						
**MSI grade**	0.007*	(1.289,4.895)	0.209		0.169		0.331	

*Data are statistically significant in terms of MSI.

### Involvement of medial/lateral pterygoid muscle only should be classified as T3, and infratemporal fossa involvement should be classified as T4

Based on our results, we propose that IFI should be separated from MLPI and be classified into a higher T stage. Alternatively, MLPI should be adjusted to a lower T stage. We further evaluated this proposal by performing two comparisons. We compared MLPI versus T3 situations of bony structures with skull base involvement and/or paranasal sinus involvement. We also compared IFI versus all other situations of T4 including intracranial involvement, cranial nerves and/or orbit involvement.

**Figure 6:**
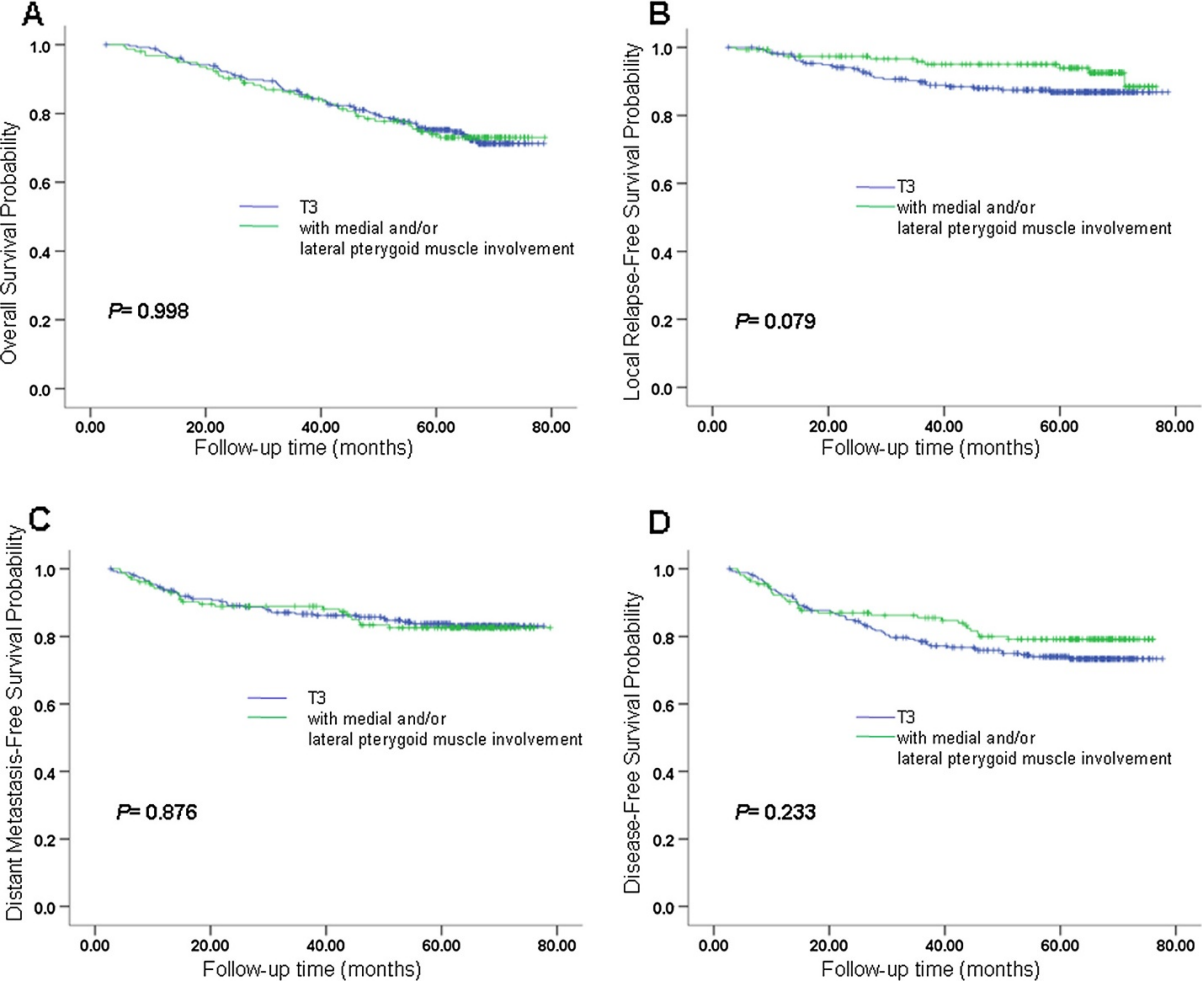
**Survival analyses for patients with T3 (UICC) VS patients with MLPI. (A)** Overall survival, **(B)** local relapse-free survival, **(C)** metastasis-free survival and **(D)** disease-free survival for patients with stage T3 and patients with medial and/or lateral pterygoid muscle involvement. MLPI, medial and/or lateral pterygoid involvement.

In the first comparison, the OS between MLPI and T3 did not show a significant difference (*P =* 0.998), nor did DMFS and DFS (*P =* 0.876, 0.223, respectively). LRFS resulted in a trend that MLPI and T3 might be different but that this result was not significant (*P =* 0.079). The survival curves of OS, LRFS, DMFS and DFS are shown in Figure 6.

**Figure 7:**
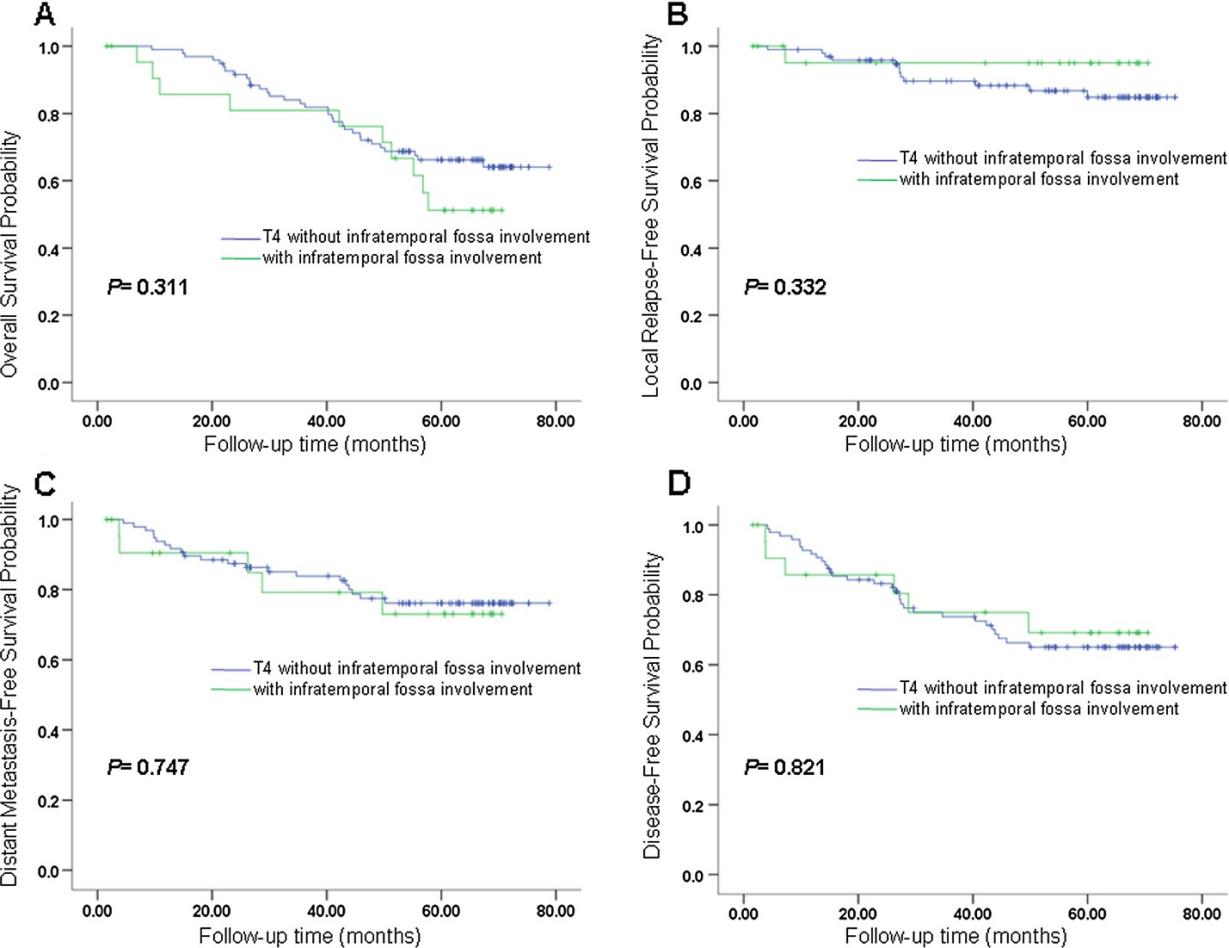
**Survival analyses for patients with T4 (UICC) without IFI VS patients with IFI. (A)** Overall survival, **(B)** local relapse-free survival, **(C)** metastasis-free survival and **(D)** disease-free survival for patients with stage T4 without infratemporal fossa involvement and patients with infratemporal fossa involvement. IFI, infratemporal fossa involvement.

The second comparison of IFI and the rest of T4 presented a similar outcome with no significant differences between these two groups in OS, LRFS, DMFS and DFS (*P =* 0.311, 0.332, 0.747, 0.821, respectively). The survival curves of OS, LRFS, DMFS and DFS are shown in Figure 7. Therefore, we validated that MLPI only should be classified as T3, and IFI should be classified as T4.

Then we carried out two comparisons in 3D-CRT/IMRT population alone, T3 (without MSI) vs. MLPI, and T4 vs. IFI, according to the analysis pattern of the entire sample. In the comparison of T3 (without MSI) and MLPI, there were 40 cases in T3 group and 20 cases in MLPI group. The OS, LRFS, DMFS and DFS between T3 and MLPI did not displayed any significant difference (P = 0.693, 0.804, 0.270, 0.754, respectively). In the comparison of T4 and IFI, there were 11 cases in T4 group and 7 cases in IFI group. The OS, LRFS, DMFS and DFS between T4 and IFI did not displayed any significant difference (P = 0.739, 0.254, 0.971, 0.441, respectively), either. Results like this might possibly indicate MLPI should be classified as T3 and IFI should be classified as T4 both in 3D-CRT/IMRT population and conventional radiotherapy population.

## Discussion

In the present study, we observed that MSI grade was an independent prognostic factor for OS and DMFS. When the tumor invades beyond the lateral pterygoid muscle and into the infratemporal fossa, it independently indicated shorter OS and DMFS. This demonstrated that grading MSI as MLPI and IFI may be a valuable prognosis indicator in NPC. The reason for the patients with IFI have a significantly higher risk of distant failure than in patients with MLPI might be a bulky primary tumor can lead to tumor invasion into the venous plexus, thereby increasing the risk of hematogenous dissemination. At the same time, MSI correlated with advanced N classifications as shown in Table 1. These results suggest that severe MSI has a potential to distant spreading. The present study also revealed that anatomic masticator space involvement was significantly associated with tumor infiltration at the base of the skull, in paranasal sinuses, intracranial, prevertebral space, and parapharyngeal space. This is consistent with the previous study by Liang et al. [21] in which local disease spread stepwise from proximal sites to distal sites in NPC is observed. Several investigators have also reported that a larger tumor volume is associated with an increased rate of tumor recurrence and poor patient survival rates [22, 23]. The reason no MSI pattern influenced LRFS or DFS might be due to the intensive use of MRI with high-quality images that allowed more accurate tumor volume recognition. Conversely, the dose distribution was improved by 3D-CRT and IMRT, which provided good local control. Consequently, MSI and some local extensions that previously influenced LRFS may be less useful with current technology.

AJCC 7th TNM staging system for NPC refers the involvement any part of the entire masticator space as T4. However, the 2008 Chinese TNM staging system defined involvement of medial pterygoid as T3 and the involvement of the lateral region of medial pterygoid in the masticator space as T4. As several versions of TNM staging systems for NPC arise, there will be additional studies attempting to determine which MSI stage is appropriate and how MSI influences survival. A study of the 2008 Chinese TNM staging system performed by Mao YP et al. [24] reported in 2009 that the risk of local failure of medial pterygoid involvement was close to the risk of local failure of intracranial extension/cranial nerve involvement and cavernous sinus involvement. The study suggested T4 should include medial pterygoid involvement. Sun Y et al. [25] demonstrated that LRFS of medial pterygoid involvement was worse than that of base of skull bony structure (T3), which is consistent with AJCC 7th staging system for NPC. Tang LL et al [10] revealed that anatomic masticator space involvement affected the OS and LRFS of patients with NPC and they recommended that anatomic masticator space involvement that includes the medial and lateral pterygoid muscles be classified as stage T4 disease.

The results of our study demonstrated that patients with IFI had a poorer overall survival compared to MLPI patients. Additionally, there were homogeneous survival rates between MLPI and T3, IFI and the rest of T4 disease. The OS between MLPI and T3 did not show significant difference, nor did IFI and T4. So we suggested that MLPI should be distributed into T3 disease and IFI distributed into T4 disease. Our findings will help to reevaluate the present TNM staging systems for NPC including the AJCC 7th and 2008 Chinese systems. Our results provide suggestions and options to improve the T staging system by classifying MLPI as T3 and IFI as T4 disease.

A new T staging system based on this study of MSI and the revised definition of T3/T4 could be used for medical management. Patients with IFI should be separately evaluated from patients with MLPI. It might be useful to identify the high-risk group from within all MSI patients to allow adjustments of treatment strategy. Additional studies are required to observe the efficacy of this modified T staging classification.

There are some limitations in the current retrospective study. One limitation was the different radiation modalities in our patient cohort and the other was the use of different chemotherapy modalities. The use of IMRT has provided encouraging results [26–28]. IMRT is the main radiation modality for NPC in our cancer center and worldwide now. But at that time (2005), due to unavoidable economic limitations, only 122 (16.4%) patients in our cohort underwent 3D-CRT/IMRT. Thus, to confirm our scientific findings, a prospective study with a relatively large cohort treated by IMRT is warranted. We believe that with the prevailing use of IMRT worldwide, some prognostic factors for NPC patients could be changed.

## Conclusions

MSI was an independent prognostic factor for OS and DMFS. NPCs invading the masticator space should be separately categorized into MLPI and IFI groups. Our data suggest that MLPI should be staged as T3 while IFI is staged as T4 in future TNM staging revisions.

## Authors’ information

Luo D-H (first author).

Yang J (co-first author).

## Abbreviations

NPC: Nasopharyngeal carcinoma
AJCC: American joint committee on cancer
MSI: Masticator space involvement
MRI: Magnetic resonance imaging
MLPI: Medial and/or lateral pterygoid involvement
IFI: Infratemporal fossa involvement
OS: Overall survival
LRFS: Local relapse-free survival
DMFS: Distant metastasis-free survival
DFS: Disease-free survival.
